# Evaluation of breeding objectives, breeding practices and reproductive performance of indigenous dairy cows in selected districts of Kaffa Zone, South West Ethiopia

**DOI:** 10.1002/vms3.1267

**Published:** 2023-09-20

**Authors:** Yakob Asfaw, Regasa Begna, Worku Masho

**Affiliations:** ^1^ Department of Animal Science Mizan – Tepi University Mizan Aman Ethiopia

**Keywords:** age at first calving, artificial insemination, calving interval, cattle, natural mating

## Abstract

**Background:**

Breeding objectives are designed to achieve targeted dairy cow production goals, which can be affected by production type, farmer preferences, environmental factors and genetic factors individually or in combination. Breeding practices, such as both controlled and uncontrolled, and artificial insemination (AI) are the tools used to obtain the desired breeding objectives. The lower reproductive performance of indigenous dairy cows affects the total milk production and calf crops that are produced during their lifetime. Designing appropriate breeding objectives and breeding practices can improve the reproductive performance of dairy cows and their overall production performance.

**Materials and methods:**

The current study was conducted with the objective of evaluating the breeding, practices and performance of indigenous dairy cattle in the south western part of Ethiopia. The districts of Gesha and Chena were purposefully chosen. The study design for the 384 household surveys was a cross‐sectional survey with a simple random sample approach. Data analysis was carried out by MS‐Excel (2010) and the general linear model procedure of SAS of 2008.

**Results:**

The current study revealed that methods of breeding were predominantly natural‐controlled mating, followed by natural‐uncontrolled mating and AI in descending order. Breeding objectives were input function, output function, sociocultural and economic functions and assets and security functions in decreasing order of rank. Reproduction performance indexes of indigenous dairy cows age at first service (3.72 ± 0.05 years), age at first calving (AFC) (4.71 ± 0.07 years), calving interval (CI) (1.58 ± 0.03 years), days open (DO) (4.26 ± 0.11 months), services per conception in natural mating (1.4 ± 0.08) and AI (2.73 ± 0.14), age of bull at maturity (4.17 ± 0.74 years), interoestrus interval (23.18 ± 0.61 days), calves crop (7.53 ± 0.22) and the life span of indigenous dairy cow (11.94 ± 0.26 years) were significant (*p* < 0.01) between two districts, whereas the values of age of bull at maturity and number of services per conception in natural mating were significant (*p* < 0.05) between districts.

**Conclusions:**

Using AI and major reproduction performances, such as AFC, CI and DO of indigenous dairy cows in the study area, were very low. Therefore, concerned bodies should intervene to improve reproduction performance through the utilization of AI techniques, with the integration of forage development activities and improvements in livestock health care.

## Graphical abstract

This study revealed methods of breeding were predominantly natural‐controlled mating, followed by natural‐uncontrolled mating and AI in descending order. The values of calving interval and days open were significantly (*p* < 0.01) higher between two districts, seasons and parities. The value of AFS and AFC were significantly (*p* < 0.01) varied between two districts.

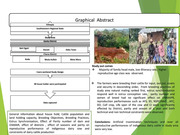

## 1 INTRODUCTION

Cattle breeds fall into two main types, which are regarded as either two closely related species or two subspecies of one species. *Bos indicus* (or *Bos taurus indicus*) cattle, commonly called zebu, are adapted to hot climates and originated in tropical parts of the world, such as sub‐Saharan Africa, India, China and Southeast Asia (Purdy et al., 2021). *Bos taurus* (*Bos Taurus taurus*), typically referred to as taurine cattle, are generally adapted to cooler climates and include almost all cattle breeds originating from Europe, the Mediterranean region and northern Asia (Marleen et al., 2014). Both species were likely present since ancient times in northern Africa and the Middle East, where both natural and human‐caused hybridization likely occurred (Upadhyay et al., 2017).

In Ethiopia, there are 37 recognized indigenous cattle breeds (DAGRIS, 2017). They serve as a source of draught power for the rural farming population, supply farm families with milk, meat and manure, serve as a source of cash income and play a significant role in the social and cultural values of society. Cattle contribute nearly all the draught power to agricultural production at the smallholder level in Ethiopia (DAGRIS, 2017). The Central Statistical Agency (CSA) (2018) report revealed that the total cattle population of Ethiopia is 65.35 million. Out of these, female cattle constitute about 55.90% and 44.10% are male cattle. Out of these, 98.2% were unimproved indigenous breeds, 1.62% were crossbreeds and 0.18% were exotic pure cattle breeds (CSA, 2017a, 2017b). From these populations, dairy cows are estimated to be approximately 12.3 million heads, and milking cows are about 6.6 million heads (CSA, 2018). Even though the country has a higher number of cattle populations, their production and reproduction performance are very low due to technical, economic and institutional constraints. Total production of milk and meat reaches 5.6 billion litres and 1.1 million tonnes, respectively. Beyond providing foods and other goods and services to the population, the livestock sector is a major contributor to export earnings, mainly through the export of live cattle and small ruminants. The annual milk yield of the country was 3.1 billion litres with a 1.64 L daily milk yield and a 6‐month average lactation length (CSA, 2018).

Kaffa zone is located in the south western regional (SWRP) state. According to the CSA (2018), the region has 5358,959 cattle population. Even though it was the home of a huge dairy cattle population, production and reproduction performance were below ideal levels. Welfare, management practices and breeding techniques were traditionally encircled with technical and non‐technical constraints. Moreover, recent biotechnology‐assisted dairy cow production, artificial insemination (AI) and standardized selection criteria were completely in their infant stages. Farmers were literally innocent and had no expectation for the mitigation of hazards, even if they occurred suddenly, and feeding was communal, private and on some occasions both on grazing land and from naturally occurring water sources. In the past, different researchers attempted to study dairy cattle management and its constraints in some parts of the country. Though the work on evaluating breeding objectives, breeding practices and performances of indigenous dairy cows have not been investigated in the south western part of Ethiopia, particularly in the Kaffa zone. Therefore, this study aimed at evaluating the breeding objectives, practices and reproductive performance of indigenous dairy cattle in selected districts of Kaffa zone.

## 2 MATERIALS AND METHODS

### 2.1 Description of study area

**FIGURE 1:**
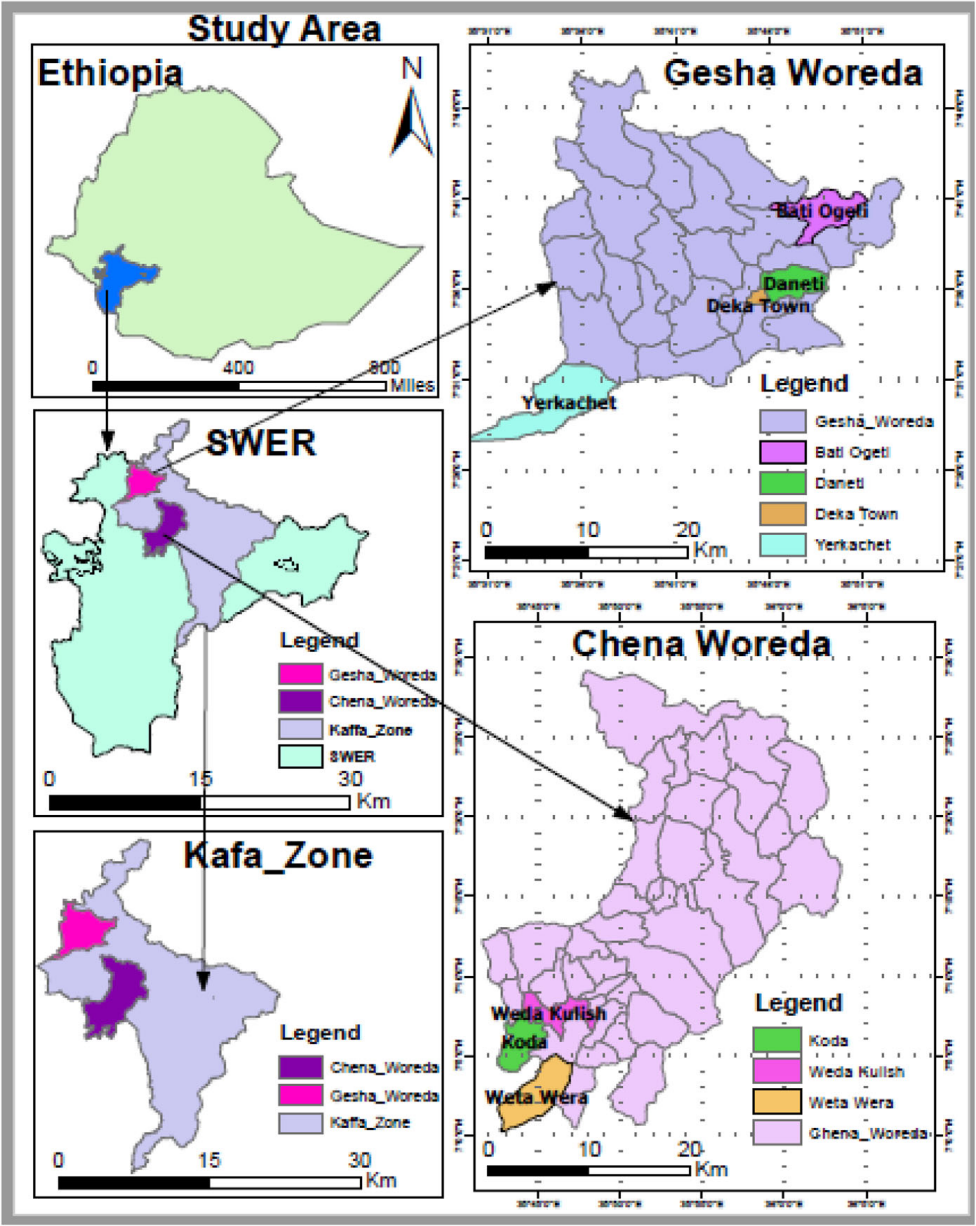
Map of study area.

South West Ethiopia Peoples Region (SWEPR) is a regional state among 11 regions found in Ethiopia. It was formed through the split of former Southern Nations Nationalities and Peoples Region (SNNPR), after conducting a successful referendum. It comprises Keffa, Sheka, Bench Sheko, Dawro, West Omo Zones and Konta Special Woreda. Kaffa zone is found between 6°24′ and 8°13′ north latitude and 35°30′ and 36°46′ east longitude. It is divided into the northern Jimma zone in the north, the Sheka Zone in the north‐west, Bench‐Sheko in the south‐west, the west Omo Zone in the south and Konta special woreda in the south‐east. It is divided into three traditional climatic zones based on height and climatic considerations. The Highland (2500–3000 masl), Midland (1500–2500 masl) and Lowland (500–1500 masl) make up 11.6%, 59.5% and 28.9% of the total area, respectively. The yearly temperature ranges from 10.2 to 27.5°C, with rainfall ranging from 100 to 2450 mL. Depending on humidity, the dense rainy season is June–September, the transitional or short rainy season is October, November and May, and the dry season is December–April (KZANRDD, 2021). As a result, in the current study, the dry season was represented by April, whereas the rainy season was represented by June. The zone is known for a mixed crop–livestock production system, which is used to produce dairy cows (KZANRDD, 2021). Therefore, two representative woredas, namely Gesha and Chena, were selected to evaluate breeding objectives, breeding practices and the reproduction performance of indigenous dairy cows (Figure 1).

### 2.2 Study design and data collection

A cross‐sectional study design was employed to evaluate breeding, practices and performances of indigenous dairy cattle.

#### 2.2.1 Sampling techniques

Aside from the districts that were specifically chosen, three kebeles from each district were picked using basic random selection approaches (six kebeles in total). Similarly, 64 farmers were chosen at random from each of the kebeles for the cross‐sectional research (a total of 384). For the monitoring project, 32 lactating cows with varying parity numbers and lactation phases were purposefully selected from survey data.

#### 2.2.2 Sample size determination

The sample size was calculated using the formula given by Thrusfield (2018).

#### 2.2.3 Sources and methods of data collection

The types of data sources were both primary and secondary. Primary data were collected from all households, focus group discussions (FGDs) and key informants by a well‐structured and pretested questionnaire, an interview and field observation. Key informants encompassed livestock and fishery development office heads, private practitioners, traders and input suppliers. Group discussions were conducted with top kebele administrators, model farmers and extension workers, with 7–10 members in each kebele. Principled enumerators who graduated from TVET and Dandi Boru Animal Science College were selected, trained and employed with the researchers’ closest follow‐up for significant and relevant data collection. Secondary data were collected from written documents of the livestock and fishery resource development department and offices, the agriculture and natural resource development department and offices, the centre of AI, epidemiology reports, veterinary anamnesis records, published journals, articles and books.

#### 2.2.4 Oestrus synchronization

The sources of information were secondary data obtained from records of AI centres and veterinary clinics for 5 consecutive years from 2017 to 2021. After rectal examination, a single shot of 5 mL of PGF2α was administered intramuscularly to those cows possessing corpus luteum and not pregnant cows. A treated cow expresses oestrus symptoms for 3–5 days. Dairy cows or heifers used for hormonal synchronization are classically selected based on their pedigree anamnesis, body conditions and body size (CWLFRDO, 2021; GWLFRDO, 2021). Inseminators checked the stage of follicles through rectal palpation when the cow was brought to the AI centre. All animals in standing oestrus are inseminated without hormone injection. A prostaglandin hormone (synchromate) PGF2α (5 mL) single‐injection protocol (Enzaprost, with a manufacture date of 02/2020 and expiration date of 02/2023) was used in the study area. Frozen semen of Holstein‐Friesian, Jersey and Boran crossbred bulls, taken from the National Artificial Insemination Center, Kality, and Addis Ababa was used for insemination. A pregnancy diagnosis test was done by rectal palpation on a growing embryo after 3 months of post‐insemination (CWLFRDO, 2021; GWLFRDO, 2021).

### 2.3 Data management and analysis

Collected data on objectives of animal breeding and breeding practices obtained from individual farmers were entered into an MS Excel spreadsheet (Excel, 2010) for data clearance, and indices were calculated to rank breeding objectives and breeding practices by the farmer according to the following formula: Index = The value of the index corresponds to the order of ranks (the highest index value, the most desired/favoured variable). Index is the sum of (*n* times number of response criteria ranked first + *n* times number of response ranked second… + number of response ranked *n*th) given for particular qualitative variables divided by the sum of responses under each rank summation of (*n* − 1 times total response ranked first +*n* − 1 times total response ranked second…+ total response ranked *n*th) for all qualitative variables (criteria) considered (Musa et al., 2006):

$$\mathrm{Index}=\frac{\Sigma \left(n\times ranked\;\mathrm{first}\right)+\left(n-1\right)\times ranked\;\mathrm{second})+\cdots +1\times ranked\;last)\;for\;specific\;trait}{\Sigma \left(n\times ranked\;\mathrm{first}+\left(n-1\right)\times ranked\;\mathrm{second}+\cdots +1\times ranked\;last\right)\;for\;all\;trait\;in\;contemplation}$$

where *n* is the number of trait ranks in concern.

Oestrus synchronization response rate and conception rate (CR) of dairy cows were calculated by using percentage and the Chi‐square test. Reproduction performance data were analysed using the general linear model (PROC GLM) procedure of SAS (2008). If there was a significant difference between means, the Tukey honestly significance test at *α* < 0.05 and *α* < 0.01 were considered significant and highly significant differences, respectively, to adjust the mean separation. The model for the analysis was Yijkl = *μ* + Ai + Sj + Pk + εijkl, where Yijklm is response variables, *μ* is the overall mean (intercept), Ai is the effect of district, Sj is the effect of parity, Pl is the effect of season and εijkl is random error.

## 3 RESULTS

### 3.1 General information of households

Household family head, age class in year and literacy rate of the study area are presented in Table 1. Males accounted for 88.8% of household heads, and the proportion of female heads was 11.2%. A greater proportion of female heads was recorded in Chena (16.7%) than in Gesha (5.7%). The female head was at the lowest benchmark in the current study. The values of age classes between 15 and 24, 55 and 64 and >65 years, life expectancy and family size in the current study were significantly (*p* < 0.05) higher for Gesha district. The current study revealed that a higher percentage of illiteracy was recorded for Chena district compared to Gesha.

**TABLE 1:** General household characteristics of study area.

Variables	Gesha	Chena	Total	*χ* ^2^	*p*‐Values
Family head (%)					
Male head	181 (94.3%)	160 (83.3%)	341 (88.8%)		0.001 (**)
Female head	11 (5.7%)	32 (16.7%)	43 (11.2%)	11.5	
Age class in year (mean ± SE)					
0–14 years	2.25 ± 0.1	2.2 ± 0.01	2.24 ± 0.1	1.58	0.812 (NS)
15–24 years	2.0 ± 0.09	1.8 ± 0.09	1.9 ± 0.06	15.15	0.004 (**)
25–54 years	1.7 ± 0.08	1.6 ± 0.08	1.6 ± 0.06	5.338	0.254 (NS)
55–64 years	0.93 ± 0.05	0.7 ± 0.05	0.8 ± 0.03	14.42	0.006 (**)
>65 years	0.74 ± 0.05	0.5 ± 0.04	0.64 ± 0.03	11.76	0.003 (**)
SP household	1.31 ± 0.08	1.11 ± 0.07	1.21 ± 0.05	3.84	0.428 (NS)
Life expectancy	69.8 ± 0.6	67.1 ± 0.46	68.5 ± 0.38	86.65	0.000 (**)
Family size	7.6 ± 0.25	6.8 ± 0.24	7.2 ± 0.18	33.248	0.007 (**)
Academic standard (%)					
Illiterate	44 (22.90%)	54 (28.10%)	98 (25.5%)	2.84	0.6 (NS)
Read and write	57 (29.7%	55 (28.60%)	112 (29.2)		
Elementary	62 (32.30%)	62 (32.30%)	124 (32.3)		
High school	26 (13.50%)	20 (10.40%)	46 (12.0%)		
College	3 (1.6%)	1 (0.50%)	4 (1.0%))		
Total LR	148 (77.1%)	138 (69.9%)	384 (74.48%)		

Abbreviations: *χ* ^2^, chi square; LR, literacy rate; NS, non‐significant (*p* > 0.05); SE, standard error; SP household, spouses per household.

*p < 0.05.

**p < 0.01.

### 3.2 Cattle population and land holding capacity

Table 2 illustrates the cattle population and land holding capacity of houses in the study area. Results showed that cows were the leading numbers with 3.31 ± 0.12 followed by calves at 2.53 ± 0.10, bulls at 1.05 ± 0.01, heifers at 0.77 ± 0.03 and steers at 0.26 ± 0.01 without significant variation between the districts. Land holdings per individual household were 2.72 ± 0.40 ha, with a significant difference (*p* < 0.05) between districts. This was due to scattered population habitat and the presence of un‐demarcated forest and grassland.

**TABLE 2:** Cattle population and household land holding capacities of study area.

Cattle population	Gesha (mean ± SE)	Chena (mean ± SE)	Total (mean ± SE)	*p*‐Values
Cows	3.52 ± 0.10	3.10 ± 0.02	3.31 ± 0.12	NS
Calves	2.74 ± 0.03	2.32 ± 0.10	2.53 ± 0.10	NS
Bulls	1.10 ± 0.07	1.00 ± 0.04	1.05 ± 0.01	NS
Heifers	0.83 ± 0.01	0.71 ± 0.02	0.77 ± 0.03	NS
Steers	0.33 ± 0.01	0.20 ± 0.00	0.26 ± 0.01	NS
Land holds (ha)	2.93 ± 0.18	2.51 ± 0.62	2.72 ± 0.40	0.026 (*)

Abbreviations: NS, non‐significant (*p* > 0); SE, standard error.

*p < 0.05.

### 3.3 Breeding objectives

In this study, the fundamental objectives were classified into four basic categories (Table 3). Breeding objective variation rose from dairy production system distinction in the study area.

**TABLE 3:** Objectives of dairy cow breeding.

	Gesha (*N* = 192)		Chena (*N* = 192)		Total
Types of breeding objectives	Index	Rank	Index	Rank	Index/*R*
Output function					0.302/second
Milk	0.86	First	0.79	First	
Meat	0.12	Second	0.2	Second	
Manure	0.02	Third	0.01	Third	
Input function					0.317/first
Traction	0.96	First	0.92	First	
Mating	0.03	Second	0.08	Second	
Assets or security function					0.130/fourth
As account	0.63	First	0.48	Second	
As buffer	0.37	Second	0.52	First	
Sociocultural and economic function					0.251/third
Income source	0.53	First	0.72	First	
Prestige	0.3	Second	0.24	Second	
Dowry	0.17	Third	0.06	Third	

*Note*: Asset, acts as a kind of savings account and durable form of storing wealth; Buffer, for astonishing jeopardies to generate immediate income restoration.

### 3.4 Breeding practices

The breeding practices of indigenous dairy cows are presented in Table 4. In this study area, natural‐controlled mating was favoured, followed by natural‐uncontrolled and AI, which ranked first, second and third, respectively. Sources of replacement for breeding bulls and heifers were from known pedigree (ancestry), and markets favoured them in decreasing rank order.

**TABLE 4:** Breeding practices of indigenous dairy cow in study area.

		Gesha (*N* = 192)		Chena (*N* = 192)	
Breeding technique		Index	Rank	Index	Rank
Natural mating	Controlled	0.498	First	0.507	First
	Uncontrolled	0.358	Second	0.286	Second
Artificial mating	AI	0.144	Third	0.208	Third
Sources of heifers and bull replacement	known pedigree	0.644	First	0.606	First
	Markets	0.356	Second	0.394	Second

### 3.5 Oestrus synchronization practices and its efficiency on dairy cow

Table 5 shows the overall efficiency of oestrus synchronization in indigenous dairy cows in the study area. The results revealed that overall oestrus response rate to a single injection of PGF2α (synchromate) was 70.5%. However, there were variations in oestrus response efficiency rates among years of inseminations, between breeds and districts. The most probable factors for the variation in oestrus response rate could be hygiene and manipulation methods of semen while processing, management habits, AI technicians, insemination time and intimated factors. The study revealed that the overall CR and fruitfulness of massive synchronization were 9.85% in study area.

**TABLE 5:** Oestrus synchronization profile.

District	Year	Semen	Synchronized	Inseminated	ERR (%)	Conceived	CR (%)
	2017	HF	188	151	80.3	13	8.60
	2018	HF	204	174	85.3	17	9.80
Gesha	2019	Jersey	231	137	59.3	10	7.30
	2020	Cross	342	209	61.1	21	10
	2021	Jersey	168	131	78.0	14	10.70
	Total		1133	802	70.8	75	9.28
	2017	HF	201	143	71.1	18	12.60
	2018	HF	349	203	58.2	24	11.80
Chena	2019	Jersey	162	123	76.0	9	7.30
	2020	Cross	241	186	77.2	15	8.10
	2021	Jersey	191	148	77.5	17	11.50
	Total		1144	803	70.2	83	10.30

*Note*: CR = conceived/inseminated (Sharifuzzaman et al., 2015); where CR stands for conception rate.

Abbreviation: ERR, oestrus response rate.

### 3.6 Effects of parity number of the dam and semen breed on OSMAI PR

As reported in Table 6, the results of the study revealed that parity of cows and breeds of semen did not affect CR (*p* > 0.05). According to the AI centre record and key informants, the major causes of failure to exhibit oestrus and conceive after oestrus synchronization and mass artificial insemination were AI technician incompatibilities with breeding technology. This was due to a lack of training and awareness, poor management of semen and ill‐handling techniques and selection of inappropriate animals. Besides chronic diseases, gynaecological disorders and the provision of long‐lasting semen were factors. Moreover, poor management, neglect and not availing cows when it revealed oestrus at the right time were listed.

**TABLE 6:** Effects of parity number of the dam and semen breed on oestrus synchronization and mass artificial insemination pregnancy rate (OSMAI PR).

	Gesha			Chena		
Variables	Inseminated	Conceived	PR	Inseminated	Conceived	PR
Parity						
Heifer	76	11	11.5	91	7	7.7%
1–2 parities	318	30	9.4	300	34	11.3%
3–4 parities	266	20	7.5	206	25	12.1%
5–6 parities	142	14	9.9	206	17	8.3%
Total	802	75	9.4	803	83	10.3%
*χ* ^2^			3.452			2.693
*p*–Value			0.327 (NS)			0.441 (NS)
Semen type						
HF	325	31	9.5%	346	42	12.1%
Cross	209	20	9.6%	186	15	8.1%
Jersey	268	24	9.0%	271	26	9.6%
Total	802	75	9.4	803	83	10.3%
*χ* ^2^			0.075			2.410
*p*‐Value			0.96 (NS)			0.3 (NS)

*Note*: ‘χ^2^’ stands for chi square.

### 3.7 Reproduction performances of indigenous dairy cow

The main indicators of reproductive performances were age at first service (AFS), age at first calving (AFC), calving interval (CI), days open (DO), number of services per conception (NSPC) in natural and AI, eligibility of bulls to serve, interservice or interoestrus intervals (IOIs), alive calf crop, voluntary waiting period (VWP), and average life span of dairy cows presented in Table 7.

**TABLE 7:** Effects of district on reproductive performances of dairy cow.

Variables	Gesha (mean ± SE)	Chena (mean ± SE)	Average (mean ± SE)	*p*‐Value
Age at first service (year)	3.47 ± 0.05	3.98 ± 0.04	3.72 ± 0.05	0.000 (**)
IOI (days)	21 ± 0.68	25.35 ± 0.77	23.18 ± 0.61	0.009 (**)
NSPC (natural mating)	1.3 ± 0.11	1.5 ± 0.11	1.4 ± 0.08	0.03 (*)
NSPC (AI)	2.7 ± 0.22	2.75 ± 0.19	2.73 ± 0.14	0.864 (NS)
Age at first calving (year)	4.44 ± 0.08	4.98 ± 0.06	4.71 ± 0.07	0.005 (**)
Days open (months)	3.83 ± 0.17	4.7 ± 0.07	4.26 ± 0.11	0.002 (**)
CI (year)	1.43 ± 0.03	1.73 ± 0.04	1.58 ± 0.03	0.006 (**)
Calf crop	8.3 ± 0.24	6.75 ± 0.29	7.53 ± 0.22	0.000 (**)
Bull puberty (year)	3.57 ± 0.11	4.76 ± 1.49	4.17 ± 0.74	0.02 (*)
Life span of cow (year)	13.1 ± 0.3	10.78 ± 0.2	11.94 ± 0.26	0.000 (**)

Abbreviations: AI, artificial insemination; CI, calving interval; SE, standard error.

*p < 0.05.

**p < 0.01.

AFS of dairy cows in the current study was 3.72 ± 0.05 years with statistically significant variation (*p* < 0.01) between districts. IOI was 23.18 ± 0.61 days with a highly significant difference (*p* < 0.01) between districts. Mating was accomplished at this moment artificially or naturally according to the preferences of farmers and the compatibility of dairy cows for service. NSPC of dairy cows in current study was 1.4 ± 0.08 for natural mating with significant difference (*p* < 0.05) between districts. NSPC in AI in current study was 2.73 ± 0.14 in study area without significant variation (*p* > 0.05) between districts. According to the key informants, survey and FGD, AI was not favoured. This was due to its limited CR, interruption of accessibility, technicians and infrastructural‐based constraints. However, the AFC for indigenous dairy cows in the current study was 4.71 ± 0.07 years with highly significant variation (*p* < 0.01) between districts. Similarly, DO recorded for indigenous dairy cows in current was 26 ± 0.11‐month highly significant difference (*p* < 0.01) between districts. VWP was unimaginative in the study area.

CI of dairy cows in the present study was 1.58 ± 0.03 years, with highly significant variation (*p* < 0.01) between districts. Current study found, the age at puberty of bulls to come up fully capacitated to inseminate was 4.17 ± 0.74 years with significant variation (*p* < 0.05) between districts. In study area, this age was believed that, after a bull attained the average puberty age (4.17 ± 0.74 years), it was allowed to serve (both mate females and responsible for traction). The results of current study revealed that calves harvested during the complete reproductive life span of cow from the onset of AFC to the beginning of menopause stage (manifested by the complete cessation of reproductive capacity) were 7.53 ± 0.22 with statistical difference (*p* < 0.01) between districts. The total life span (the longevity of life to stay as alive on this planet) reported in present study was 11.94 ± 0.26 years, with highly significant variation (*p* < 0.01) between districts.

### 3.8 Effects of season of the year and parity number on reproductive performances

Elements of reproductive performance that are greatly prone to seasonal alteration within a year are IOI, NSPC, CI and DO. The results of this study revealed that the season of the year can affect IOI in the dry and wet seasons of the year, with highly significant variation (*p* < 0.01) between districts (Table 8). Moreover, NSPC both in natural mating and AI were affected (*p* < 0.01). The impact of season also can extend to the DO of dairy cows. According to the current study result publicized, there was a highly significant difference in DO between dry and wet seasons of the year (*p* < 0.01). Similarly, indigenous dairy cows in the study area had significantly (*p* < 0.01) higher CI for the dry season of the year.

**TABLE 8:** Season and parity effects on reproductive performances of indigenous dairy cow in study area.

Fixed factors	IOI (mean ± SE)	NSPC (NM) (mean ± SE)	NSPC (AI) (mean ± SE)	DO (mean ± SE)	CI (mean ± SE)
Season					
Dry	26.2 ± 0.9	1.87 ± 0.22	3.47 ± 0.22	4.67 ± 0.08	1.72 ± 0.05
Wet	21.36 ± 0.58	1.08 ± 0.06	2.2 ± 0.16	4.02 ± 0.16	1.49 ± 0.4
Total	23.18 ± 0.61	1.38 ± 0.11	2.68 ± 0.16	4.26 ± 0.11	1.58 ± 0.3
*p*‐Value	0.000 (**)	0.000 (**)	0.000 (**)	0.004 (**)	0.001 (**)
Parity number					
First	27.09 ± 0.96	1.82 ± 0.26	3.27 ± 0.27	4.68 ± 0.1	1.75 ± 0.06
Second	20.6 ± 0.68	1.13 ± 0.09	2.13 ± 0.24	3.83 ± 0.19	1.39 ± 0.03
Third	22.86 ± 0.81	1.29 ± 0.16	2.79 ± 0.26	4.39 ± 0.18	1.65 ± 0.04
Total	23.18 ± 0.61	1.38 ± 0.11	2.68 ± 0.16	4.26 ± 0.11	1.58 ± 0.03
*p*‐Value	0.000 (**)	0.025 (*)	0.013 (*)	0.005 (**)	0.000 (**)

Abbreviations: AI, artificial insemination; CI, calving interval; DO, days open; IOI, interoestrus interval; NM, natural mating; NSPC, number of services per conception; SE, standard error.

*p < 0.05.

**p < 0.01.

Here it was revealed that first parity had the longest days to cycle oestrus than the second and third parties, with a highly significant difference (*p* < 0.01) by exposing parity determination on reproduction efficiency. Dissimilar to the parity linear increment, NSPC in natural and artificial mating increased abruptly in the sequence of second, third and first parities, with significant variation (*p* < 0.05) in both conditions and techniques of mating. DO was affected by parity number (*p* < 0.01) with the highest DO in the first parity than in the second and third parities in months. In a similar way, CI was prone to the impacts of parity number and found a highly significant effect (*p* < 0.01) between parity number and CI of indigenous dairy cows in the study area.

### 3.9 Constraints of dairy production in study area

Table 9 discusses the major constraints on dairy cattle production in the study area. For the keenest clarity, constraints are categorized into two categories: technical and non‐technical challenges. Top prioritized constraints in study area were poor genetic make‐up, prevalence of diseases, feed scarcity and water scarcity from the technical side. Moreover, the lack of infrastructure (roads, electricity, pure water, clinics and services), the lack of access and the high cost of improved dairy heifers/cows and the lack of research and information exchange between the government and NGOs were predominating non‐technical delayers. According to FGD, key informants and survey results, reasons for culling dairy cows from herds in study area were low reproduction capacities and production performances, non‐infectious diseases like fractures, disabilities and genetic defects like poor pedigree inheritance, sterility, the lack of sexual libido, anoestrus, anatomical defects of the reproduction tract causing excessive dystocia and pendulous udder due to loose median suspensory ligament.

**TABLE 9:** Leading constraints of dairy production in study area.

	Gesha (*N* = 192)		Chena (*N* = 192)		Total
Challenges	Index	Rank	Index	Rank	Index/*R*
Technical constraints					0.351/first
Poor genetic make‐up	0.331	First	0.317	First	
Diseases	0.243	Second	0.182	Third	
Culling loss	0.185	Third	0.077	5th	
Feed and water scarcity	0.136	Fourth	0.279	Second	
Climatic factors	0.105	Fifth	0.145	4th	
Non‐technical constraints					0.649/second
Lack of information exchange	0.120	Fifth	0.236	Second	
Lack of infrastructures	0.250	First	0.154	Fourth	
Lack of education and consultation	0.170	Third	0.162	Third	
Inadequate extension and training services	0.085	Sixth	0.060	Sixth	
Loss of market linkage	0.156	Fourth	0.131	Fifth	
High cost of improved dairy heifers/cows and bull	0.219	Second	0.256	First	

## 4 DISCUSSIONS

### 4.1 General information of households

The higher male family head recorded for current study was friendly with Wondatir and Mekasha (2014), who reported 86.7% and 13.3% male‐ and female‐headed households in the northern highland area. However, current study result for a male‐headed household was higher than Bekele et al. (2015) and Jobir and Yohannes (2021) who reported 77.78% and 80% in Dangila and Abaya woredas, respectively. Even though the current study accessed different age hierarchies, it targeted productive ages 25–54 years. This stratum is the most responsible for themselves, different aspects of the country (CSA, 2018), and comparatively less prone to dementia (Center for Disease Control [CDC], 2019). The family size of household was higher than the estimated national family size of 4.6 family members in Ethiopia and the world's average of 4.0 family size per household (WPDS, 2020). Friendly result was reported by Haile et al. (2012) and Misganu (2019), having 7.13 ± 0.24 and 7.34 family sizes, respectively. Abebe et al. (2021) also reported that 6.42 ± 2.28 and 6.18 ± 2.17 in urban and peri‐urban dairy production systems, respectively, which was lower than current study result. The life expectancy of indigenous dairy producers in study area was higher than national report of 65.5 years (The World Bank, 2018) and 67.07 years (ELE, 2021). Most probably, it was due to the less consumption of fabricated food which can predispose for obesity linked diseases and the presence of enough oxygen supply in rural areas enhancing erythropoiesis to maintain cells to live longer (Pyrkov et al., 2021). Misganaw et al. (2021) testified that illiterate 46.2%, read and write 6.18%, elementary school 58.48%, junior school 29.2%, high school above 12.02% in peri‐urban dairy production system. Abebe et al. (2021) also reported a result in northern highland area antagonizing the current result in little bit. Illiterate (10.14%), primary school (43.48%), secondary education (36.23%) and above secondary school (10.14%) were reported in Mekele dairy cow producers (Idesa & Aman, 2021). Dachu and Kefele (2021) reported that 30% were illiterate and 67% and 3% were educated in elementary and secondary, respectively. The literacy rate reported in this study exceeds the literacy rate report of sub‐Saharan Africa, which was 64.0% but much lower than global literacy rate, which was 86.3% (UNESCO, 2019).

The households in the current study were proportionally educated than 73.6% and 64% illiterates in trans‐human and sedentary production system in Enderta district (Tsadikan. 2012). In addition, it was better than illiterate 53.3%, primary school 36.7%, secondary school 6.7% and higher 3.3% in Yabello (Nurye Gebeyehu, 2017). Better educated households were reported by Belay and Janssens (2014), in Jimma urban dairy production system. Generally, dairy farmers need to get more education and extension service for efficient dairy cow production in the country as stated by Gwandu et al. (2018) in Tanzania.

### 4.2 Cattle population and land holding capacity

The current result recorded on cattle population was a little bit disagreed with the report of Fissha and Deng (2021) cows 4.75 ± 0.19, heifers 1.76 ± 0.13, bulls 1.68 ± 0.11 and calves 1.65 ± 0.07 and steers 1.07 ± 0.09 in lowland parts of Ethiopia. Abebe et al. (2021) also reported average dairy cow was 4.48 ± 1.85, heifers 1.89 ± 1.0 and 1.66 ± 0.47 bulls. The number dairy cow per household was higher than 1.04 ± 0.89 the value reported by Leyla (2016) at the Agarfa district. However, lower cattle population as calves 1.28 ± 0.04, heifer 1.39 ± 0.04, bull 1.35 ± 0.05 and lactating dairy cow 1.25 ± 0.04 were reported in Jimma zone dairy producers (Misganu, 2019). Dairy production system, the prevalence of epidemic and endemic diseases, accessibility of land, purpose of production and agro‐ecological factors were responsible for livestock and dairy cow population variation (Fissha and Deng, 2021; Abebe et al., 2021).

The land holding capacity of households reported in current study was higher than previous South Nation Nationality and Peoples Region (SNNPR) (0.49 ha), Oromia region (1.15 ha) and 1.09 ha of Amhara region (DMoFA, 2020). It could be an element within the range of 1.5–4 ha in the Gambella region (Yaynshet et al., 2010). Gatwech (2012) reported 3.0 and 2.18 ha in urban and mixed crop–livestock production systems in the same region. Extremely low 0.0067 and 1.46 ha of land was reported in urban and peri‐urban production systems, respectively (Misganaw et al., 2021).

### 4.3 Breeding objectives

The breeding objectives of dairy producers in the study area agree with the report of Fissha and Deng (2021), who ranked milk first, meat second, asset third and dowry fourth in a mixed dairy production system. Fantahun and Admasu (2017) revealed food security, living assets, nutrition, economic sources, sociocultural merit and employment as friendly results for the current study. Kefena et al. (2011) reported that milk production, the sustainability of the herd and draft purposes were high rented objectives. Production performances, income generation, savings accounts, aesthetics, and dowry were appealing objectives as friendly report for the current study result (Hirwa et al., 2017). Traction power, food sources, economy source, employment and sociocultural functions were reported by disagreement with the current study result (Ledetu, 2019).

### 4.4 Breeding practices

In order to boost the production of milk and milk products and to meet the growing demand for these goods, it is essential that breeding practices be acceptable. This will ensure the long‐term viability of the dairy industry (Kemer et al., 2021). The breeding practices of farmers in the study area were agreeable with the report of Gondadaw et al. (2015) who conducted study on indigenous dairy cattle in Northern Amhara. Similarly, Gebremichael (2015) and Gebeyew et al. (2016) reported natural mating followed by AI in the Tigray region and Dawa Chefa district, respectively. Asrat et al. (2015) reported agreeable result in the Wolaita Sodo zone. Disobediently, Abebe et al. (2021) and Demissu et al. (2014) reported that natural mating was less preferred than AI. As it was reported by Kiros (2019), dairy farmers in the study area have remained loyal to natural mating because breeding bulls are accessible and adopted problem, semen is limited, conception failures and higher NSPC in AI due to incorrect heat detection, too early or too late insemination, dystocia, foetal oversize and technician incompatibility with reproductive technology.

### 4.5 Oestrus synchronization practices and its efficiency on dairy cow

The result reported on the efficiency of oestrus synchronization in the current study is similar to that reported by Bainesagn (2015) who reported 72.3% of the oestrus rate in the West Shoa Zone, Oromia Region. Disobediently, Debir (2016) in Sidama and Samuel et al. (2015) in West Gojjam reported 90% and 88.9% oestrus, respectively. Studies in Awassa Dale milk shed by Girmay et al. (2015) and around Wukrokilte Awulaelo district indicated that the rate of oestrus response in a single injection of prostaglandin protocol at the farmer level was 91.3%. Wubneh et al. (2021) reported 91.2% for OSMAI in Ari district. CR and the fruitfulness of massive synchronization in the study area are comparable with Wubneh et al. (2021) for indigenous breeds in the Ari district with 11.02% CR. On the other hand, Fantahun and Admasu (2017) observed 24.69% pregnancy rate in the Mizan Aman area of South West Ethiopia. Similarly, Azage et al. (2015) reported an average CR of 27.1% at the national and 33.3% at regional level (SNNPR). Similarly, findings of Samuel et al. (2015) reported 70.4%, 78.2% and 71.5% of CR for hormone‐treated Holstein‐Friesian, Jersey crosses and local cows/heifers in the Amhara region. Linearly, Detalem (2015) reported CRs of Holstein‐Friesian, Begait local and non‐descript local cows/heifers in the Tigray region were 38.4%, 39.7% and 37.7%, respectively. According to Wubneh et al. (2021), the main reasons for failure and a low pregnancy rate include improper timing of insemination, a failure to identify oestrus in time, poor feeding management, the skill of the producers and the inseminator, quality semen and improper handling.

### 4.6 Effects of parity number of the dam and semen breed on OSMAI PR

The effect of parity number and artificially inseminated cows of the current study was contrary to reports of Samuel et al. (2015) who revealed an increasing trend of pregnancy rate as the parity of cows increased. However, it was in line with the findings by Bainesagn (2015) and Debir (2016). The findings of Yeshimebet et al. (2017) in the North Shoa Zone showed that high pregnancy rates were obtained in the double injection of PGF2α treatment (63.1%) than in animals treated with one shot protocol 55.8% (*p* < 0.05). Analogously, reasons for the failure of the OSMAI campaign in Oromia, Amhara, Tigray and SNNP regions were technician inefficiency, poor quality of semen, feed problems, an inappropriate season of AI and low awareness of farmers (Tegegne et al., 2016). However, an agreeable result was reported by Misganaw (2019), from the Jimma zone with current study.

### 4.7 Reproduction performances of indigenous dairy cow

AFS was similar to 3.81 years of Kereyu breed (Shiferaw, 2006). But, the value was longer than 2.72 years of Friesian (Mengistu et al., 2016), 8–10 months for European dairy cows (Belay et al., 2012), 38.10 ± 0.47 months for local breeds in the Gondar zone (Ayeneshet et al., 2018). Contrarily, the current study result was shorter than 3.933 years of Fogera breed (Damitie et al., 2015). Similarly, 4.21 years of indigenous breed around lowland of Ethiopia (Fissha and Deng, 2021), Ogaden breed having 4.07 years (Kassahun et al., 2015) and 3.445 ± 1.020 years for local breed in Siltie zone (Bayesa & Eyob, 2021) and 53 months (Guta, 2021). The heifer's reproductive and productive activities begin with the AFS signals, which also have an impact on the female's reproductive and productive life span through their impact on her lifetime calf crop. Due to the cow's extended, non‐lactating, unproductive phase over several months, a significant delay in reaching sexual maturity may result in a significant economic loss (Tiruneh & Taddie, 2016).

IOI was equivalent to the result of 23 days reported by Blavy et al. (2018). Remnant et al. (2018) reported 28 days, which is more longer time, and Greenham et al. (2019) revealed IOI can be prolonged from 11 to 31 days within its range of the current findings. The result of NSPC of dairy cows in current study was larger than Barka breed, Holstein Friesian and their ¾ cross with Fogera breed cow with 1.11, 1.4 and 1.3, respectively, in the central highland of Ethiopia (Adisu & Zewdu, 2021). Contrarily, Ayeneshet et al. (2018) stated 1.68 ± 0.60 NSPC. In its analogous result, numbers of services per conception vary with the breed and 1.3–1.5 in exotic breeds and 1.4–2.8 in indigenous dairy cow was reported (Guta, 2021).

AFC for indigenous dairy cow of the present study was longer than national indigenous cow AFC of 4.417 years (Galmessa & Fita, 2019). Zelalem et al. (2011) reported *B. indicus* heifers reach puberty at older ages than *B. taurus* heifers HF and Borana crossbred was obtained at 34.6,33.7, 33.3 and 33.96 months in 50% (F1), 75% (F2), 87.5% (F3)and ≥93.75% (F4) blood levels, respectively. The findings were shorter than Kassahun et al. (2015) who reported 4.99 years of AFC for the Ogaden breed. The difference in the nutritional condition and management practices for dairy cows may account for the AFC variation (Kemer et al., 2021). According to studies of Gebreyohannes et al. (2019), extended AFC leads in high milk yields during the first lactation but lower lifetime output due to fewer calves being born.

DO recorded for indigenous dairy cow was comparatively longer than 2.53 months for Pure HF and pure Boran (Zelalem et al., 2011). However, the result was shorter than 4.53 months of cross 7/8 Friesian and 1/8 Boran, 4.3 months for cross of ¾ Friesian and ¼Boran (Mengistu et al., 2016). Contrarily, Ayeneshet et al. (2018) reported 9.72 ± 0.40 months. VWP was unimaginative for cows not only in the study area but also in 37 identified breeds throughout the country (DAGRIS, 2017). DOs have an impact on lifetime productivity, generation times and annual genetic gain (Tadesse, 2006).

CI of dairy cow in the present study was similar to the value of 1.50 year reported by Shiferaw (2006) for the Kereyu breed. But the findings were longer than 1.219 year of the Arsi breed (Kgaudi et al., 2018), and 1.15 year of the Friesian breed (Mengistu et al., 2016). Disobediently, the result of this study was better than extreme edges 4.51 years Begait breed (Mezgebe et al., 2017), 2.16 years of the Horro breed (Kassahun et al., 2015) and 2.08 years of average indigenous Cattle breed at national level (Ulfina & Lemma, 2019) and 18.38 ± 1.05 months for local cow in Wollo (Demeke, 2020). Guta (2021) reported CIs of zebu breeds usually vary from 12.2 to 26.6 months within the range of the result of current study result lays. The extended CI may be caused by the dam's age, genetics, the availability of food and the calving season (Ayalew and Asefa, 2013). The result of age at puberty of bull present study was higher than the value of 27.3 months for crossbreed reported and 42.2 months for local breed bull to attain full maturity. Teweldemedhn (2016) Terefe (2010) and Agere et al. (2012) reported 42 ± 6, 43.32 ± 0.96 and 46.56 ± 0.06 months for Begait, Mursi and Horro breed bulls, respectively, to attain age at maturity. Similarly, ages at puberty of bulls were 36.7 and 29.3 months for local and crossbred, respectively, reported by indicating types of breed and agro‐ecology significantly affect age at bull maturity (Amanuel, 2019). Climatic factors, variety of breeds and management practices were responsible for the inconsistency of age to attain puberty (Agere et al., 2012). Another factor that hinders the development of favourable conditions for puberty is disease (Hassan et al., 2020). *B. indicus* (indigenous cattle) in the tropics and subtropics are thought to reach puberty between the ages of 16 and 40 months (Tiruneh & Taddie, 2016).

Calves harvested during the complete reproductive life span of cow from the onset of AFC to the beginning of menopause stage (manifested by complete cease of reproductive capacity) were comparable with Solomon et al. (2011) reported 7.3 for Boran breed and Teweldemedhn (2016) reported 7 ± 1 for Begait breed indigenous dairy cows. Disobediently, Guta (2021) reported three to four live calf crops, Damitie et al. (2015) reported 4.94 ± 0.17 for Fogera, Agere et al. (2012) 6.46 ± 0.13 for Horro breed, Chali (2014) 7.0 ± 0.2 for Arsi breed. Similarly, current study showed a higher calf yield than three to four calves of average indigenous cow at national level (Galmessa & Fita, 2019). Contrarily, Demeke (2020) reported 9.59 ± 0.49 calves of the local breed of dairy cow in Wollo which was far away from the result of current study.

The total life span (the longevity of life to stay alive on this planet of the earth) reported in present study was found in the range of 11–13 years at national level reported by Galmessa and Fita (2019). Comparably, Solomon et al. (2011) reported 12.7 for Boran breed dairy cows. Higher age was declared by Agere et al. (2012) reported for Horro 13.67 ± 0.31 years, Demeke (2020) 16.21 ± 0.27 and 14.07 ± 0.23 years for indigenous cow and with their crosses, respectively. Contrarily, Damitie et al. (2015) reported 4.94 ± 0.17 for Fogera breed, and Teweldemedhn (2016) reported 11.0 ± 0.8 for Begait breed dairy cow lower than current study result. Age at puberty, first calving age, and CI all affect a cow's lifetime production, as do the cow's genetic make‐up, health state and management and feeding practices (Legesse, 2016).

### 4.8 Effects of season of the year and parity number on reproductive performances

Similar results were reported by Guta (2021) on the effect of the season of the year and parity number for NSPC, CI and DO and in all conditions, the variation was highly significant. Similarly, Anwar et al. (2017) reported that the season of calving has significant impact on reproduction performances of dairy cows. This was due to any environmental hardship and inaccessibility of feeds and health cares for dairy cow depressing performance capacities. Similarly, Kiros (2019) reported a significant effect of parity on NSPC and other reproduction performances. Furthermore, Ayalew and Asefa (2013) reported that factors associated with negative energy balance caused from malnutrition and feed scarcity have been considered causes of reproductive failure, lower CRs, longer CIs and an increased incidence of silent heat. NSC might be lower or greater depending on the need for accurate and prompt heat detection and insemination (Gebreyohannes et al., 2019).

### 4.9 Constraints of dairy production in study area

The major constraints of dairy production in the study area agreeably with the report of Fissha and Deng (2021) who reported major constraints, such as poor genetics, diseases, feed and water shortage, scarcity of services and clinics were hindering factors for pastoral and mixed crop dairy production system. Constraints can vary based on agro‐ecology, management factors and production system. Shortage of feed (43%), health problem (37%) and water security (20%) were major challenges affecting dairy cattle production and productivity in Abaya Woreda (Jobir & Yohannes, 2021). Similarly, the shortage of land, high feed price, feed shortage, disease problems and market fluctuations were the main constraints found at 84.8%, 81.8%, 75.75%, 69.69%, and 62.12%, respectively, in north western Ethiopia (Abebe et al., 2021). Haile et al. (2012) reported prioritized constraints were shortage of feed, limited space for proper housing and waste disposal and disease incidence in Hawasa. Odero‐Waitituh (2017) for Kenya and Gillah et al. (2012) for tropical dairy production reported that the shortage of feed was the leading constraint. To enhance the dairy sector in study area, constraints must be mitigated (Lokuruka, 2016) and appropriate interventions must be sought by government, non‐government and any concerned actors (Baliyan & Gosalamang, 2016). As friendly result, Hirwa et al. (2017) reported poor health, old age, infertility, high calf mortality, slow growth, small offspring, to avoid inbreeding, bad conformation, unfavourable colour and bad body condition. Similarly, disease, low milk yield, ageing, injury and infertility were leading factors for culling (Idesa & Aman, 2021).

## 5 CONCLUSIONS

The current study revealed that household family size was above the national and international estimated averages. The breeding practices were predominantly natural‐controlled mating, followed by natural‐uncontrolled, and AI in descending order. Breeding objectives were for input function, output function, sociocultural and economic function and for assets and security function in descending order. The study revealed that overall reproduction performances of indigenous dairy cows are very low due to technical and non‐technical constraints. Even though indigenous dairy cows were the powerhouse of opportunities, welfare abandonment and poor management besides constraints made them deprived of production and reproduction aptitudes under ideal standards.

## AUTHOR CONTRIBUTIONS

Yakob Asfaw contributed to designing the study, performing the study, analysing and interpreting the data and writing the manuscript. Regasa Begna Roba contributed to designing the study, supervising the study and writing the manuscript. Worku Masho Bedane contributed to designing the study, supervising the study and writing the manuscript.

## CONFLICT OF INTEREST STATEMENT

The authors declare no conflicts of interest.

## FUNDING INFORMATION

No funding was obtained for this study.

## ETHICS STATEMENT AND CONSENT TO PARTICIPATE

Animal care and ethical issues were carefully evaluated and approved for the experiment (1956ET‐12/2020) by Mizan – Tepi University, College of Agriculture and Natural Resources Ethics Committee. Directive 2010/63/EU of the European Union guidelines (2010) concerning the treatment and use of animals in research and development purposes were employed.

### PEER REVIEW

The peer review history for this article is available at https://www.webofscience.com/api/gateway/wos/peer‐review/10.1002/vms3.1267.

## Data Availability

Data used and analysed for this study are available from the corresponding author on reasonable request.
